# The Effects of Prednisone/Ketoprofen Administration in Association with Amoxicillin Clavulanate Following Periodontal Surgical Therapy in Patients with Severe Chronic Periodontitis

**DOI:** 10.3390/medicina57050447

**Published:** 2021-05-04

**Authors:** Lidia Sfetcu, Andreea Cristiana Didilescu, Cristian Vlădan, Octavian Dincă, Daniela Miricescu, Gabriela Băncescu, Alexandru Bucur, Laura Carina Tribus

**Affiliations:** 1 Department of Microbiology, Faculty of Dental Medicine, “Carol Davila” University of Medicine and Pharmacy, 020032 Bucharest, Romania; gabriela.bancescu@umfcd.ro; 2 Department of Embryology, Faculty of Dental Medicine, “Carol Davila” University of Medicine and Pharmacy, 050474 Bucharest, Romania; 3 Faculty of Dental Medicine, “Carol Davila” University of Medicine and Pharmacy, 010221 Bucharest, Romania; cristi.vladan@umfcd.ro (C.V.); alexandru.bucur@umfcd.ro (A.B.); 4 Department of Oral and Maxillofacial Surgery, “Dan Theodorescu” University Hospital of Oral and Maxillofacial Surgery, 010221 Bucharest, Romania; 5 Department of Biochemistry, Faculty of Dental Medicine, “Carol Davila” University of Medicine and Pharmacy, 050474 Bucharest, Romania; daniela.miricescu@umfcd.ro; 6 Department of Gastroenterology, Bucharest Emergency University Hospital, “Carol Davila” University of Medicine and Pharmacy, 020032 Bucharest, Romania; laura.tribus@umfcd.ro

**Keywords:** periodontitis, saliva, cortisol, amoxicillin, ketoprofen, prednisone

## Abstract

*Background and Objectives*: The aim of this study was to evaluate and compare the effects of two different anti-inflammatory drugs (ketoprofen and prednisone) combined with an antibiotic (amoxicillin + clavulanic acid) and periodontal surgery on dental and periodontal parameters in patients with severe chronic periodontitis. In addition, salivary stress expressed by cortisol levels was assessed. *Materials and Methods*: An interventional study was performed on 22 periodontal subjects and 19 clinical healthy controls. The patients were divided in four groups, depending on treatment planning, as follows: eight patients received prednisone and antibiotherapy, associated with surgical periodontal therapy; seven patients received ketoprofen and antibiotherapy, associated with surgical periodontal therapy (group II); seven patients received only prednisone. Periodontal healthy patients underwent routine scaling and polishing. Bleeding on probing (BOP), dental mobility and salivary cortisol (ng/mL) were assessed before and after treatment. The means and standard deviations for the salivary cortisol levels (SCLs), dental and periodontal parameters were calculated for all groups using each patient as a unit of analysis. *Results*: Data analyses showed that the two different anti-inflammatory drugs associated with or without surgical therapy were efficient on inflammation periodontal parameters (BOP, dental mobility). Prednisone treatment alone was associated with a significant decrease of SCLs between pretreatment and post-treatment. *Conclusions*: In the present study, the effects of either of the anti-inflammatory drugs on inflammation evolution and salivary stress were comparable in patients undergoing antibiotherapy and surgical periodontal therapy.

## 1. Introduction

Periodontal disease is a common inflammatory disease mainly caused by subgingival colonizing microorganisms that induce the host’s immune-inflammatory response [1]. Due to the inflammatory nature of periodontal diseases, some authors suggested that the progression of the disease might be influenced by administrating steroidal or nonsteroidal anti-inflammatory drugs (SAIDs, NSAIDs) [2,3]. It has been previously shown that NSAIDs and SAIDs administered in patients with periodontal disease had an effect on lowering the degrees of periodontium inflammation [4,5,6].

Among the risk factors, it has been reported that the average age associated with periodontal disease occurrence is 40 [7]. Sex is another risk factor associated with periodontal damage; the frequency of severe periodontitis was associated with males [8]. Smoking is considered a risk factor for developing periodontal disease; it has been demonstrated that smokers have a 2.5 to 3.5 times greater risk of severe periodontal attachment loss [9]. Poor oral hygiene and dental caries presence are also considered important risk factors in the prevalence of periodontal disease [10]. An increased body mass index (BMI) may increase the risk for the development of severe periodontitis [11,12]. Patients with a low socioeconomic status had a significantly higher number of aggressive periodontitis, as compared to those with a high socioeconomic status [13,14].

More recently, there has been a great interest in the role that stress plays on the hypothalamus-pituitary-adrenal axis and its implications in periodontal disease [3]. Chronic stress stimulates the hypothalamus-pituitary-adrenal cortex axis, which leads to an increase of blood and saliva cortisol levels. Cortisol is a stress hormone that causes the activation of antistress and anti-inflammatory mechanisms such as the inhibition of lymphocytes formation, inducing lymphatic tissue hyperplasia, an inhibition effect on the proliferation of fibroblasts in the inflammatory granulation tissue and the suppression of some preinflammatory cytokines [15]. Furthermore, some studies showed a positive correlation between the level of oxidative stress and the concentration of free salivary cortisol. Increased levels of cortisol have been found in patients with various forms of periodontitis and anxiety [16,17].

The purpose of the study was to evaluate and compare the effects of nonsteroidal (ketoprofen) and steroidal (prednisone) anti-inflammatory drugs associated with periodontal surgery and antibiotherapy with amoxicillin and clavulanic acid on periodontal parameters, in patients with severe chronic periodontitis. In addition, a secondary aim was to assess the variations of salivary cortisol levels before and after treatments.

## 2. Materials and Methods

### 2.1. Subjects

An interventional study was conducted between June 2017 and March 2018. The study was approved by the Ethics Committee of the Carol Davila University of Medicine and Pharmacy, Bucharest (No. 128/29.06.2017) on 29 June 2017. Twenty-two subjects with severe chronic periodontitis and 19 clinically healthy subjects were recruited from a dental private office in Bucharest. Subjects under the age of 18, with systemic diseases [18], medication allergies, pregnancy, anti-inflammatory or antibiotic drugs administration in the last six months were excluded from the study.

According to the study protocol, surgery took place in two stages: the first intervention was a maxillary open flap surgery, and the second intervention was a mandibular open flap surgery. The surgery interventions were programmed with a five-day interval, and drugs administration was done for 10 days in order to include both stages.

The patients were allocated to treatment arms as follows: eight patients received SAIDs (prednisone 5 mg/oral/1 tablet per day, 10 days) and antibiotherapy (amoxicillin 875 mg + clavulanic acid 125 mg/oral/1 tablet, two times a day, 10 days), associated with open flap surgery (group I); seven patients received NSAIDs (ketoprofen, sustained release form, 150 mg/oral/1 tablet per day, 10 days) and antibiotherapy (amoxicillin 875 mg + clavulanic acid 125 mg/oral/1 tablet, two times a day, 10 days), associated with open flap surgery (group II); seven patients received only SAIDs (prednisone 5 mg/oral/1 tablet per day, 10 days) (group III).

Nineteen periodontal healthy patients underwent dental prophylaxis procedures, that is, routine scaling and polishing; this group served as the control group regarding the salivary cortisol measurement (group IV).

### 2.2. Medical Records

Demographic data, including age, sex, weight and height, level of education, profession, medical history, medication used and smoking habits (recorded as smoker/nonsmoker) were collected. Patients’ self-reported height (in meters) and weight (in kilograms) were used to calculate the BMI (kg/m^2^) using the standard formula. The dental chart included information about brushing frequency, auxiliary methods for dental hygiene and professional cleaning.

### 2.3. Oral Examination

A clinical examination was performed on all present teeth, excluding third molars. The same trained examiner assessed the dental and periodontal parameters (LS). Patients’ dental health status was evaluated using the index DMFT/S (Decay, Missing, Filling on tooth/surface), an epidemiological index accepted by WHO (World Health Organization) [19]. The following periodontal parameters were measured at six interproximal sites for each tooth: bleeding on probing (BOP), probing pocket depth (PPD) and clinical attachment loss (CAL). The BOP index was calculated by adding up all the bleeding areas (marked with 1) multiplied by 100 and dividing the product by the total number of surfaces. PPD (the distance from the gingival margin to the bottom of the pocket) and CAL (the distance from the cement-enamel junction to the bottom of the pocket/sulcus) were measured using a conventional periodontal probe CP-C MM 3.5 (Medesy, Maniago (PN), Italy) before treatment [20]. Averages of these assessments were computed per tooth, per patient, and the average per group was subsequently performed. Dental mobility was clinically assessed for each tooth by holding it firmly between two metal objects and applying movements in different directions. The degree of dental mobility was noted as follows: normal mobility–grade 0; mobility in the transverse plane <1 mm–grade 1; mobility in the transverse plane >1 mm–degree 2; mobility transversely and vertically–grade 3 [21]. BOP and mobility were evaluated before and one month after periodontal therapy. CAL and PPD measurements were performed before treatment on the groups I, II and III because of their use for enclosing the patients into a degree of periodontal disease. Severe periodontitis was diagnosed according to the clinical case definitions proposed by the Center for Disease Control and Prevention [22]. Participants enrolled in this study signed a written informed consent that included the avoidance of any self-treatment throughout the duration of the study.

### 2.4. Salivary Cortisol Assessment

The patients were recommended to collect saliva in the morning, between 9 and 11 a.m., before brushing teeth, smoking, eating or drinking [23]. Saliva was collected in sterile containers on the day of periodontal treatment and one month after treatment. A quantitative salivary cortisol (ng/mL) determination was performed using a colorimetric immuno-enzymatic kit with serial number DSNOV20 from NovaTec Immundiagnostica GmbH (Dietzenbach, Germany). The samples were stored at −20 °C before processing, according to the manufacturer’s instructions. Using an Elisa microwell plate reader, the absorbance was read at 450 nm.

### 2.5. Statistical Analysis

Data distributions were expressed as means, medians, standard deviations, intervals and percentages, as appropriate. Based on the data distribution, intergroup analyses were performed using an unpaired *t*-test and Mann–Whitney test. Similarly, intragroup analyses were done using a paired *t*-test and Wilcoxon test. We performed statistical analyses using Stata/IC 16 (StataCorp. 2019. Stata Statistical Software: Release 16. College Station, TX: StataCorp LLC).

## 3. Results

### 3.1. Medical Records and Risk Factors Assessment

The socio-demographic aspects are presented in Table 1. Apart from residency, the features were similar among groups.

Although the mean values of the DMFT and DMFS indexes were higher in the study groups, no statistically significant differences were recorded (Table 2).

### 3.2. Preintervention Periodontal Assessments

CAL and PPD measurements were performed before treatment at six distinct points, the averages of these assessments were computed per tooth, per patient, and the average per group was subsequently performed (Table 3).

### 3.3. Pre- and Postintervention Clinical Assessments

#### 3.3.1. BOP Evaluations

In all three groups, there was a significant decrease of the BOP values after treatment (*p* < 0.05) (Table 4).

#### 3.3.2. Mobility Evaluations

The data analysis obtained from patients receiving prednisone (group III) showed that the number of teeth with mobility 0 post-treatment increased after one month as compared to the pretreatment stage. A statistically significant difference was obtained in the two groups that received surgery combined with medication (groups I and II) (Table 5).

### 3.4. Salivary Cortisol Assessments

**Figure 1:**
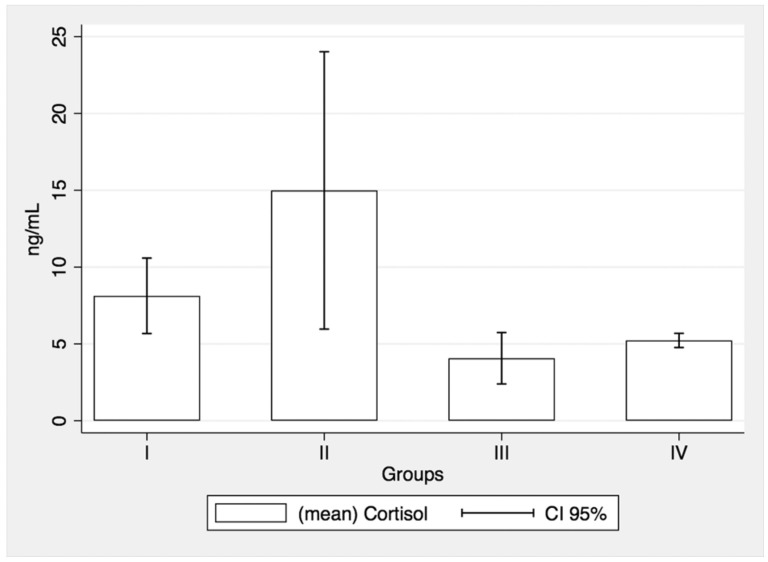
Pretreatment salivary cortisol values.

**Figure 2:**
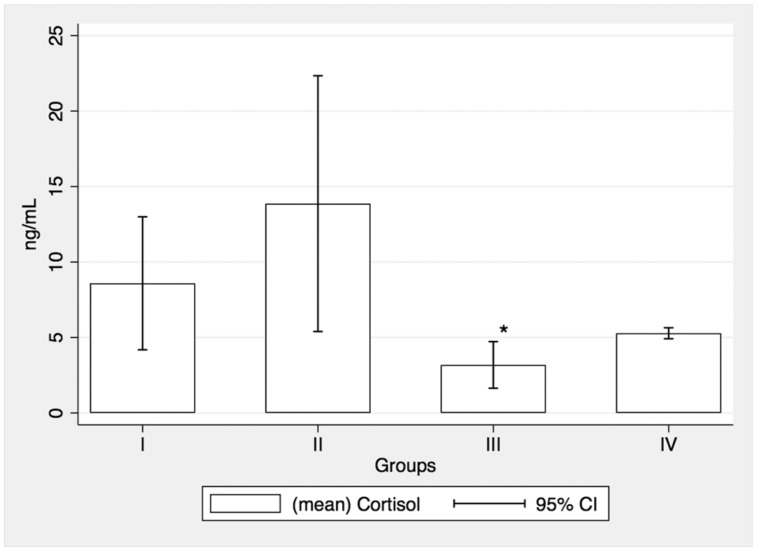
Post-treatment salivary cortisol values; * statistical significance.

No significant differences of SCLs between pre- and post-intervention were recorded in groups I (Mean 1: 8.13+/−2.81; Mean 2: 8.59+/−5.05), II (Mean 1: 14.99+/−9.34; Mean 2: 13.86+/−8.77) and IV (Mean 1: 5.22+/−0.12; Mean 2: 5.28 +/−0.35). However, steroidal treatment alone (group III) was associated with a significant decrease of SCLs between pretreatment (Mean 1: 4.06+/−1.87) and post-treatment (Mean 2: 3.18+/−1.72); t (6) = 2.55, *p* = 0.043 (Figure 1 and Figure 2).

## 4. Discussion

The main results of the present study showed a comparable effect of steroidal (prednisone) and nonsteroidal (ketoprofen) anti-inflammatory drugs associated with open flap surgery on periodontal parameters (BOP, dental mobility) and also on salivary stress expressed by SCLs.

Following this study, which evaluated 22 patients with severe chronic periodontitis, it was found that in all three intervention groups the presence of gingival bleeding decreased in a statistically significant way after all types of interventions. A clinical study retrieved from the literature assessed BOP in two groups of patients with periodontitis, one systemically healthy and one using SAIDs for general chronic inflammation, and found comparable results [24]. On the other hand, some clinical studies evaluating NSAIDs’ effects on inflammation parameters in patients with chronic periodontitis showed a significant BOP reduction [25,26]. Other previous studies evaluated NSAIDs’ effects on marginal periodontium, being performed without clinical evaluations of periodontal inflammation. Paraclinical parameters (e.g., prostaglandin E2), which are known to be involved in some events associated with inflammation in periodontal disease, were assessed. Assessments showed a reduction after NSAIDs’ administration [27,28,29,30].

Regarding dental mobility, our study showed a mobility reduction in the two groups that received surgery combined with medication (groups I and II). A study made in 2004 on the changes in tooth mobility after surgical procedures showed that in each category of teeth included in the study there was a progressive increase of tooth mobility with the peak on the seventh day, followed by a decrease in the next three months [31]. Dental mobility represents the loss of dental support, which is a host’s immune-inflammatory response to bacteria, and it can be a way of assessing the extent of gingival or periodontal inflammation [32]. The literature abounds in studies that evaluate the effects of NSAIDs on bone. All the studies evaluated bone loss through a radiographic evaluation concluding that, after administrating NSAIDs, bone loss was delayed or reduced [33,34,35]. On the other hand, a study made on bone loss in patients with long-term SAIDs therapy suggested that profound marginal periodontal bone loss did not seem to be countered by long-term SAIDs treatment [36].

In addition to the clinical data collected from pre- and postoperative patients, saliva was collected to determine the salivary cortisol level. Increased levels of salivary cortisol may be associated with the evolution and severity of periodontal disease [37]. Following the assessment of salivary cortisol of patients in group III, there was a significant reduction in the amount of salivary cortisol between the two assessments. It is noted that pretreatment salivary cortisol values were low in this group, even if the periodontal damage was severe. The low values of salivary cortisol can be explained by the fact that the patients came from rural areas; the degree of stress caused by the job was probably lower, but the nutrition was also different. The chances of periodontitis occurring in close relationship with a stressful job are six times higher than of having it occur when one does not have a stressful job [1]. It is possible that for patients in group III the major cause of developing the condition would be represented by poor oral hygiene and less by the environment of origin and living conditions [38]. Additionally, the difference in salivary cortisol values between the initial and post-treatment calibration may be due to prednisone administration, which has the effect of reducing oxidative stress [39]. The difference between group III and groups I and II in terms of cortisol values after treatment may also be due to the fact that patients in groups I and II were additionally subjected to the stress of the surgery. The cortisol measurements in patients subjected to surgical procedures in dentistry (group I and II) showed higher cortisol levels at the end of the treatment, comparable with other previous results [40,41]. Periodontal degradation has recently been reported as having a positive correlation with SCLs [42].

Considering the small number of patients and relatively short duration of the study, future studies involving larger groups of patients and repeated long-term monitoring will be able to add more robust information to the field. Due to salivary cortisol’s circadian rhythm [43], this study’s results must be interpreted while taking into account this limit.

## 5. Conclusions

In the present study, the effect of prednisone on periodontal inflammation evolution was comparable to that of ketoprofen in combination with periodontal surgery and antibiotherapy. BOP showed a significant variation between the two evaluation stages, a decrease occurring for all three intervention groups with patients diagnosed with severe chronic periodontitis.

The effect of the treatments on the dental mobility was more pronounced in the groups where surgery was performed.

Regarding salivary stress, the effects of prednisone and ketoprofen were comparable in the patients undergoing surgical periodontal therapy. Treatment with prednisone without associated surgical therapy can be effective in reducing short-term gingival inflammation.

## Figures and Tables

**Table 1:** Baseline characteristics of all groups.

	Groups *	I (*n* = 8)	II (*n* = 7)	III (*n* = 7)	IV (*n* = 19)
Variable					
Age Mean (SD **; range)		43.25 (12.8; 24–59)	45.28 (9.7; 28–54)	46.28 (16.76; 26–72)	41.1 (9.84; 28–68)
Male (%)		62.50	42.86	57.14	57.89
Smokers (%)		37.50	42.86	57.14	57.89
Higher education (%)		50	57.14	42.86	73.57
Urban resident (%)		87.50	85.71	28.57	100
BMI Mean * (SD **; range)		28.43 * (9.5; 17.71–47.34)	24.97 * (4.16; 19.60–30.48)	23.78 * (3.59; 19.15–27.77)	26.09 * (5.45; 19.49–41.87)
No scaling per year (%)		37.5	71.43	57.14	36.84
Two times/day brushing (%)		100	100	71.43	89.47
Using auxiliary methods to brushing (%)		0	14.29	0	15.79

B.M.I., body mass index. * Group I: eight patients, prednisone + amoxicillin, clavulanic acid + open flap surgery; Group II: seven patients, ketoprofen + amoxicillin, clavulanic acid + open flap surgery; Group III: seven patients, prednisone; Group IV: 19 periodontal healthy patients, dental prophylaxis procedures. ** Standard deviation.

**Table 2:** DMFT and DMFS assessments for all groups.

Groups *	DMFT	DMFS
	Mean (SD **; Range)	Mean (SD **; Range)
I (*n* = 8)	12.5 (6.45; 3–22)	49 (27.27; 10–86)
II (*n* = 7)	14 (6.70; 4–21)	60.14 (37.86; 14–105)
III (*n* = 7)	11.57 (5.56; 3–18)	44.14 (26.56; 6–78)
IV (*n* = 19)	11.65 (4.20; 3–19)	38.35 (22.84; 7–90)

DMFT, Decay, Missing, Filling on tooth; DMFS, Decay, Missing, Filling on surface. * Group I: eight patients, prednisone + amoxicillin, clavulanic acid + open flap surgery; Group II: seven patients, ketoprofen + amoxicillin, clavulanic acid + open flap surgery; Group III: seven patients, prednisone; Group IV: 19 periodontal healthy patients, dental prophylaxis procedures. ** Standard deviation.

**Table 3:** CAL and PPD assessments for the intervention groups.

Groups *	CAL (mm)	PPD (mm)	Site
	Mean (SD **; Range)		
I (*n* = 8)	3.96 (0.62; 3.25–5.35)	3.49 (0.56; 2.92–4.82)	155.37 (12.68; 126–168)
II (*n* = 7)	3.94 (0.23; 3.49–4.25)	3.69 (0.35; 3.06–4.16)	145.71 (24.49; 109–180)
III (*n* = 7)	3.82 (0.99; 2.68–5.44)	3.17 (0.81; 2.16–4.86)	159.57 (23.10; 126–191)

CAL, clinical attachment loss; PPD, probing pocket depth. * Group I: eight patients, prednisone + amoxicillin, clavulanic acid + open flap surgery; Group II: seven patients, ketoprofen + amoxicillin, clavulanic acid + open flap surgery; Group III: seven patients, prednisone. ** Standard deviation.

**Table 4:** BOP assessments for the intervention groups.

Groups *	BOP		
	Pre-Treatment	Post-Treatment	Significance
	Mean (SD **, Range)		
I (*n* = 8)	37.10 (9.91; 24.07–58.33)	4.63 (3.06; 0–8.33)	*p* = 0.011 ***
II (*n* = 7)	49.52 (13.63; 29.16–74.24)	8.15 (6.37; 0–20.37)	*p* = 0.018 ***
III (*n* = 7)	43.05 (16.25; 19.79–71.42)	27.29 (11.44; 9.52–43.58)	*p* = 0.022 ***

* Group I: eight patients, prednisone + amoxicillin, clavulanic acid + open flap surgery; Group II: seven patients, ketoprofen +amoxicillin, clavulanic acid + open flap surgery; Group III: seven patients, prednisone. ** Standard deviation. *** Statistically significant, Wilcoxon test.

**Table 5:** Mean mobility assessments for the intervention groups.

Groups *			Tooth Mobility		
		Grade 0	Grade 1	Grade 2	Grade 3
		Mean (SD **; Range)			
I (*n* = 8)	Pretreatment	5.25 (4.29; 0–15)	10.37 (5.17; 1–14)	6.37 (2.82; 1–9)	4.37 (3.70; 0–13)
	Post-treatment	12.87 (4.51; 6–12)	7.12 (1.53; 5–10)	2.37 (2.78; 0–9)	0.25 (0.66; 0–2)
II (*n* = 7)	Pretreatment	8.71 (4.06; 1–15)	9.85 (4.58; 3–13)	3.85 (2.64; 1–8)	1.57 (0.90; 0–3)
	Post-treatment	14.57 (6.18; 6–12)	5.85 (3.48; 3–16)	1.14 (8.83; 0–2)	0
III (*n* = 7)	Pretreatment	16.71 (5.77; 8–26)	5.71 (2.05; 2–8)	2.71 (3.14; 0–8)	1.14 (1.24; 0–3)
	Post-treatment	17.28 (6.27; 8–28)	6.28 (3.75; 0–13)	1.57 (2.38; 0–7)	1.14 (1.24; 0–3)

* Group I: eight patients, prednisone + amoxicillin, clavulanic acid + open flap surgery; Group II: seven patients, ketoprofen + amoxicillin, clavulanic acid + open flap surgery; Group III: seven patients, prednisone. ** Standard deviation.

## Data Availability

The data presented in this study are available from the corresponding authors upon reasonable request.
